# InterTransViewer: a comparative description of differential gene expression profiles from different experiments

**DOI:** 10.18699/VJGB-23-119

**Published:** 2023-12

**Authors:** А.V. Tyapkin, V.V. Lavrekha, E.V. Ubogoeva, D.Yu. Oshchepkov, N.A. Omelyanchuk, E.V. Zemlyanskaya

**Affiliations:** Institute of Cytology and Genetics of the Siberian Branch of the Russian Academy of Sciences, Novosibirsk, Russia Novosibirsk State University, Novosibirsk, Russia; Institute of Cytology and Genetics of the Siberian Branch of the Russian Academy of Sciences, Novosibirsk, Russia Novosibirsk State University, Novosibirsk, Russia; Institute of Cytology and Genetics of the Siberian Branch of the Russian Academy of Sciences, Novosibirsk, Russia; Institute of Cytology and Genetics of the Siberian Branch of the Russian Academy of Sciences, Novosibirsk, Russia; Institute of Cytology and Genetics of the Siberian Branch of the Russian Academy of Sciences, Novosibirsk, Russia; Institute of Cytology and Genetics of the Siberian Branch of the Russian Academy of Sciences, Novosibirsk, Russia Novosibirsk State University, Novosibirsk, Russia

**Keywords:** transcriptome, data integration, auxin, ethylene, Arabidopsis thaliana L., транскриптом, интеграция данных, ауксин, этилен, Arabidopsis thaliana L.

## Abstract

Meta-analysis of transcriptomic data from different experiments has become increasingly prevalent due to a significantly increasing number of genome-wide experiments investigating gene expression changes under various conditions. Such data integration provides greater accuracy in identifying candidate genes and allows testing new hypotheses, which could not be validated in individual studies. To increase the relevance of experiment integration, it is necessary to optimize the selection of experiments. In this paper, we propose a set of quantitative indicators for a comprehensive comparative description of transcriptomic data. These indicators can be easily visualized and interpreted. They include the number of differentially expressed genes (DEGs), the proportion of experiment-specific (unique) DEGs in each data set, the pairwise similarity of experiments in DEG composition and the homogeneity of DEG profiles. For automatic calculation and visualization of these indicators, we have developed the program InterTransViewer. We have used InterTransViewer to comparatively describe 23 auxin- and 16 ethylene- or 1-aminocyclopropane-1-carboxylic acid (ACC)-induced transcriptomes in Arabidopsis thaliana L. We have demonstrated that analysis of the characteristics of individual DEG profiles and their pairwise comparisons based on DEG composition allow the user to rank experiments in the context of each other, assess the tendency towards their integration or segregation, and generate hypotheses about the influence of non-target factors on the transcriptional response. As a result, InterTransViewer identifies potentially homogeneous groups of experiments. Subsequent estimation of the profile homogeneity within these groups using resampling and setting a significance threshold helps to decide whether these data are appropriate for meta-analysis. Overall, InterTransViewer makes it possible to efficiently select experiments for meta-analysis depending on its task and methods.

## Abstract (ru)

В настоящее время в связи со стремительным ростом количества полногеномных экспериментов по изучению изменения экспрессии генов в различных условиях все более широкое распространение получают методы метаанализа транскриптомных данных из разных экспериментов, так как интеграция данных может обеспечить большую точность в выявлении генов-кандидатов и позволяет тестировать новые гипотезы, которые невозможно было проверить в отдельных исследованиях. Для повышения информативности такой интеграции необходимо оптимизировать подбор экспериментов. В настоящей работе мы предлагаем набор количественных показателей для всестороннего сравнительного описания транскриптомных данных. Эти показатели легко могут быть визуализированы и интерпретированы. Они включают в себя количество дифференциально экспрессирующихся генов (ДЭГ), долю специфических (уникальных) ДЭГ в каждом наборе данных, попарное сходство экспериментов по составу ДЭГ, оценку однородности профилей дифференциально экспрессирующихся генов. Для автоматического вычисления и визуализации этих показателей мы разработали программу InterTransViewer. Мы применили InterTransViewer для сравнительного описания транскрипционных ответов на обработку фитогормонами у модельного растения Arabidopsis thaliana L., взяв в анализ 23 единообразно обработанных профиля дифференциальной экспрессии генов в ответ на ауксин и 16 профилей дифференциальной экспрессии, индуцированных этиленом или его предшественником – 1-аминоциклопропановой кислотой. Мы продемонстрировали, что комплексное рассмотрение характеристик отдельных профилей ДЭГ в контексте результатов попарных сравнений профилей по составу ДЭГ позволяет позиционировать эксперименты в контексте друг друга, оценивать тенденцию к их интеграции или сегрегации, генерировать гипотезы о влиянии весомых нецелевых факторов на исследуемый транскрипционный ответ. В результате это дает возможность выделять потенциально однородные группы экспериментов. Последующий анализ однородности этих групп профилей с помощью процедуры ресемплинга и установления порога уровня значимости помогает принять решение о целесообразности использования этих данных для метаанализа. В целом InterTransViewer позволяет эффективно формировать выборки экспериментов в зависимости от задачи и методов метаанализа. об-

## Introduction

Analysis of differential gene expression under various conditions is one of the most promising approaches for studying the genetic regulation of traits (Stelpflug et al., 2016; Tello-Ruiz et al., 2016). The rapid increase in the number of experiments on whole-genome profiling of gene expression under different conditions and the availability of their results in functional genomics databases such as Gene Expression Omnibus (GEO) (Clough, Barrett, 2016) or BioStudies (Sarkans et al., 2021) open a wide space for comparative analysis of experimental results from different studies aimed at generalizing them across studies using meta-analysis (Cahan et al., 2007; Rung, Brazma, 2013; Keel, Lindholm-Perry, 2022). Such an approach allows not only to extract the most robust differentially expressed genes (DEGs) (Freire-Rios et al., 2020), but also to increase sample size to identify weak patterns (Bairakdar et al., 2023) or to test hypotheses that could not be investigated in individual studies (Sudmant et al., 2015; Winter et al., 2019).

For successful integration, data must meet several criteria (Cahan et al., 2007; Rung, Brazma, 2013; Yu, Zeng, 2018). First of all, experiments should be characterized according to the established minimum requirements for transcriptome experiments (Brazma et al., 2001; Brazma, 2009). In addition, the experiments should investigate similar hypotheses on the effect of the same factor. At the same time, one should avoid or correct the so-called batch effect, when non-target factors (biological characteristics of the object, experimental conditions, sample preparation protocol, choice of the data acquisition platform, etc.) affect the results of the experiment.

Simple data filtering by experimental conditions does not always ensure optimal selection of data for meta-analysis. On the one hand, a significant non-target factor may not be mentioned in the metadata, and formal matching of experimental conditions does not always rule out a batch effect. On the other hand, the results of experiments performed under nonidentical conditions can be fairly well matched. Comparative description of transcriptome data from different experiments allows to optimize the choice of data and methods for data preprocessing. However, no standard has yet been developed for this procedure, and there is a significant lack of appropriate software tools, especially for graphical presentation of the results. For example, MetaQC program used for microarray quality assessment evaluates six quantitative metrics: (1) reproducibility of co-expressed groups of genes across experiments, (2) consistency of the co-expression pattern of known genes with databases of metabolic and signaling pathways (i. e. involvement of genes in the same process); (3–4) accuracy of detecting the enrichment of the DEG group in Gene Ontology terms (i. e., gene involvement in processes, association with cellular components or molecular functions) and their consistency across experiments; (5) accuracy of detection of known biomarkers; (6) consistency of DEG ranking between transcriptomes (Kang et al., 2012). However, MetaQC does not visualize these metrics, and some of the quality metrics rely on external databases and known markers rather than internal features of expression profiles, which can obscure insufficiently studied processes and complicate analyses for non-model species.

Another program, ViDGER, designed to simplify the interpretation of data from RNA sequencing experiments, provides a wide range of visualizations but does not offer a convenient means to compare DEG profiles (McDermaid et al., 2019). NetworkAnalyst 3.0 emphasizes the reconstruction of protein-protein interaction networks, but also provides the ability to visually compare gene lists using interactive heat maps, enrichment networks, Venn diagrams, and chord diagrams (Zhou et al., 2019).

In this paper, we propose a set of easily visualized and interpreted indicators for a comprehensive comparative description of DEG profiles. These indicators characterize individual differential expression profiles, their pairwise similarity, and their tendency to integrate or segregate. To automatically calculate and visualize these indicators, we developed the InterTransViewer program, which we applied to comparatively describe transcriptional responses to auxin (23 DEG profiles from 16 studies) and ethylene (16 DEG profiles from 8 studies) in Arabidopsis thaliana.

## Materials and methods

Characteristics of individual differential expression profiles. In each hormone-induced transcriptome, we composed the DEG list. Next, we estimated (1) the number of DEGs, (2) the ratio of DEGs specific only for this DEG list to the total number of DEGs in the list, and (3) the ratio between the proportion of specific DEGs in the DEG list and the proportion of the transcriptome DEGs in the joint DEG list from all transcriptomes under study:

**Formula. 1.**
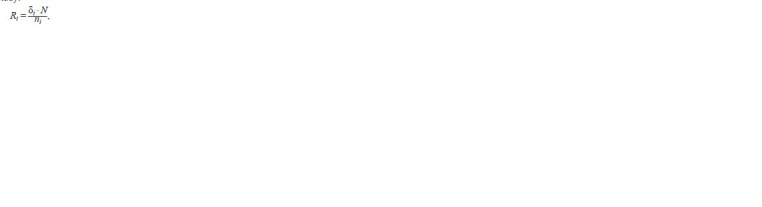
Formula. 1

where Ri is the ratio between two proportions for the DEG list i, δi is the proportion of DEGs specific for the DEG list i, ni is the number of DEGs in the DEG list i, N is the number of DEGs in the joint DEG list for all transcriptomes under study. The calculated indicators are graphically represented using mirrored histograms. Together with the metadata, they provide a first approximation for the similarity of DEG profiles and enable identification of potential outliers. For example, a too small or a too large number of DEGs or a high R value that do not correlate with specific experimental conditions or biological properties of the sample may indicate the influence of an unknown non-target factor or poor data quality.

Pairwise comparison of differential expression profiles by DEG composition. If a smaller DEG list is nested within a larger DEG list, and the deviation of the size of each DEG list from the mean is insignificant or correlated with specific experimental conditions or biological properties of the sample, we consider the results of the two experiments to be consistent. Therefore, to assess the similarity of any two DEG lists, we calculated the similarity index I as follows:

**Formula. 2.**
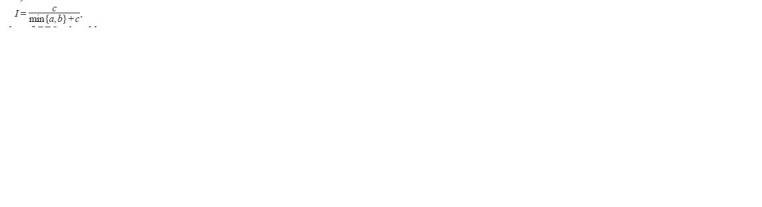
Formula.2

where c is the number of DEGs shared between the DEG lists, a is the number of DEGs present in the first and absent in the second DEG list, b is the number of DEGs present in the second and absent in the first DEG list. Thus, the similarity index I reflects the proportion of shared DEGs in the smaller DEG list. The similarity index can take values from zero to one, with zero corresponding to the absence of shared DEGs in two DEG lists, and one corresponding to full nesting of one DEG list in the other. DEG list similarity matrices are visualized as a heatmap, on the basis of which one can not only infer the similarity of expression profiles by DEG composition, but also identify individual groups of the most similar experiments

Clustering of differential expression profiles. The similarity matrix described in the previous section compares the DEG lists without considering fold changes in gene expression. To identify groups of similar differential expression profiles, we used hierarchical clustering based on a matrix of Euclidean distances in the log2-transformed space of fold changes in gene expression (log2FC), without considering statistical significance of fold changes. To allow comparison of transcriptional response profiles from different experiments, fold changes were normalized to the range in each experiment and standardized for each gene beforehand. Hierarchical clustering was performed with the Bclast function from the shipunov v.1.17.1 (https://CRAN.R-project.org/package=shipunov) package, using the Ward.D2 method based on minimizing the sum of squares of the Euclidean distances between each object of the cluster and the cluster centroid.

Quantitative evaluation of homogeneity by DEG composition within a group of profiles. Let A be the set of genes identified as DEGs in at least one of the m analyzed DEG lists, and the number of these DEGs be | A | = N. The set А includes (1) DEGs, changes in the expression level of which in a given sample of m DEG lists are determined predominantly by the influence of a target factor, and (2) genes, changes in the expression level of which are significantly affected by non-target factors. Obviously, if we calculate the value of Nk for a subsample of k DEG lists (k < m) and then, adding one DEG list at a time to this subsample, calculate the values of Nk+i , then the value of Nk+i should not decrease as the i value grows. In this case, the more heterogeneous the set of DEG profiles (the more DEG lists formed under the influence of different non-target factors it contains), the stronger the growth of the Nk+i value will be.

**Fig. 1.**
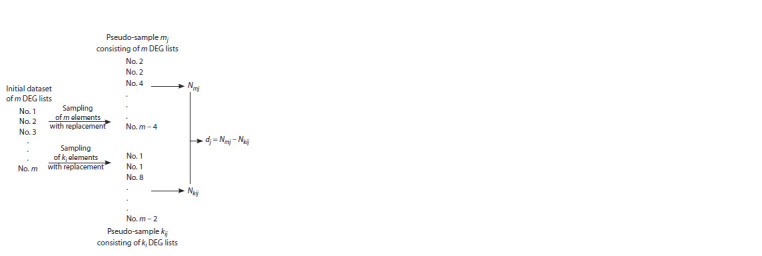
The procedure for creating two pseudo-samples, each consisting of m and ki (ki < m) DEG lists selected randomly with replacement, and determining the difference dj between the number of DEGs in at least one of the m and ki DEG lists (Nmj and Nkij , respectively). The subscript j denotes the serial number of the pseudo-sample. The operation was repeated 5000 times ( j ∈ ℕ, j = [1; 5000]), thus generating a distribution of d values, which allowed to assess the significance of the difference of N values in the pseudo-samples. This procedure was repeated for each value of ki (k1 = m – 1; ki+1 = ki – 1, where i ∈ ℕ, i = [1; m – 1]).

Using resampling, we created m – 1 sets of pseudo-samples of DEG lists: in one set i (i∈ℕ, i = [1; m – 1]), each pseudosample consisted of ki < m DEG lists (k1 = m – 1, ki+1 = ki – 1), to estimate at what value of ki there would be a meaningful decrease in Nki compared to Nm. To form a single pseudosample, from the original set of DEG lists consisting of m elements, we randomly selected ki DEG lists with replacement (Fig. 1). For each pseudo-sample, we determined the number of genes Nkij identified as DEGs in at least one of the ki DEG lists (index j denotes the number of the pseudo-sample in the same set). Simultaneously, we created a pseudo-sample of m DEG lists and calculated the corresponding value of Nmj, then calculated the difference dj = Nmj – Nkij (see Fig. 1). As a result of 5000 iterations ( j∈ℕ, j = [1; 5000]), a variational series of these differences was generated. The confidence interval was determined using the percentile method (Rousselet et al., 2021). If a significant difference between Nm and Nki was observed at some values of ki, the analyzed set of profiles was considered heterogeneous. The distribution of d values was visualized as a histogram

Implementation of the InterTransViewer program. The InterTransViewer program is implemented as an R script (v.4.1.2) and is available at (https://github.com/al-t1/Inter TransViewer0/). InterTransViewer takes as input a table, in which the first column contains one grouping variable (gene identifiers, IDs) and each subsequent pair of columns contains log2-transformed gene expression fold change values (logFC) and the corresponding adjusted p values for each individual experiment. If the user preprocessed the raw transcriptome data independently, such a table can be assembled using InterTransViewer’s DEGweave function, which combines the results generated by the limma topTable function (for microarrays) and/or the DESeq2 results function (for RNAseqs). It is advisable to perform preprocessing of raw data as uniformly as possible for each technology platform, and that the design of each experiment should include at least two biological replicates in both control and treatment trials. During the quality control step, it is recommended to pay particular attention to the data variation among replicates: for example, to employ the plotMDS function from the limma package for microarrays (Ritchie et al., 2015); fastQC and fastp for raw RNA-seq data (http://www.bioinformatics.babraham.ac.uk/ projects/fastqc/; Chen et al., 2018) and to utilize the plotPCA function from the DESeq2 package for a count matrix (Love et al., 2014). It is essential that all differential expression profiles reflect the action of a single target factor. Technically, a DEG list is suitable for analysis with InterTransViewer if it has at least one DEG at the selected significance level, but it is recommended to have at least 10 DEGs in the DEG list.

The calculation of indicators for the comparative description of DEG profiles and their visualization are implemented as functions described in the InterTransViewer documentation. For example, the number of DEGs, the fraction of experimentspecific DEGs and the Ri ratio for all experiments can be obtained using the DEGsummary function and visualized as bar charts using the TotalSpecPlot and RmetricPlot functions. The GetSimMatrix function allows to obtain the similarity matrix I. The DE_bootstrap function allows resampling as described above. Hierarchical clustering is performed using the DE_clustering function. Finally, InterTransViewer generates a wide range of output data. For each transcriptome, two tables are generated containing a DEG list and a list of transcriptome-specific DEGs, both supplemented with the corresponding logFC and p-adj values.

InterTransViewer also outputs the following: the total list of genes that are DEGs in at least one experiment with the number of experiments, in which the gene is a DEG; the summary table generated by the DEGsummary function, and the corresponding histograms; the similarity matrix I, and the corresponding heatmap; the dendrograms obtained by clustering; tables and diagrams with resampling results to assess the homogeneity within groups of DEG lists.

Transcriptome datasets from publicly available sources. We collected all publicly available transcriptomic data on the treatment of A. thaliana with phytohormones auxin, ethylene, their precursors, or synthetic analogues. From those, we have selected transcriptomes of whole seedlings or individual organs of wild-type plants, in which hormone treatments were complemented by control experiments (mock treatment or no treatment). To allow subsequent comparative analysis, we performed uniform preprocessing of the raw data. Microarray data were downloaded from the GEO database (https://www. ncbi.nlm.nih.gov/geo/). RNA-seq data were extracted from the NCBI Sequence Read Archive (SRA) (https://www.ncbi. nlm.nih.gov/sra/). The genome sequence of A. thaliana and its annotation (TAIR 10) were downloaded from Ensembl Plants (https://plants.ensembl.org/index.html, release 52).

All microarray experiments found were performed using the ATH1 platform. Raw microarray data normalization and DEG calling were performed with the limma v.3.52.4 package (Ritchie et al., 2015). FastQC v.0.11.9 (http://www. bioin formatics.babraham.ac.uk/projects/fastqc/) was used to assess the quality of RNA-seq data. Illumina reads were trimmed and quality filtered with fastp v.0.23.2 (Chen et al., 2018) using the following parameters: -q 20 -u 30 -5 -3 -W 4 -M 20. The reads were aligned to the A. thaliana genome with HISAT2 v.2.2.1 (Kim et al., 2019). SOLiD reads were aligned to the genome using TopHat (Kim et al., 2013). To quantify the number of uniquely mapped reads, we used the summarizeOverlaps function from the GenomicAlignments R package v.1.30.0 (Lawrence et al., 2013) with A. thaliana genome annotation. DEGs were called using the DESeq2 package v.1.34.0 (Love et al., 2014). For each dataset (both microarray and RNA-seq), we applied the Benjamini–Hochberg multiple hypothesis testing correction (Benjamini, Hochberg, 1995) to control the false discovery rate (FDR) for DEG calling. To detect DEGs, we used an FDR threshold of 0.05. As a result, we obtained 23 and 16 DEG lists for auxin and ethylene treatment, respectively. Each list contained at least 300 DEGs (see the Table).

**Table 1.**
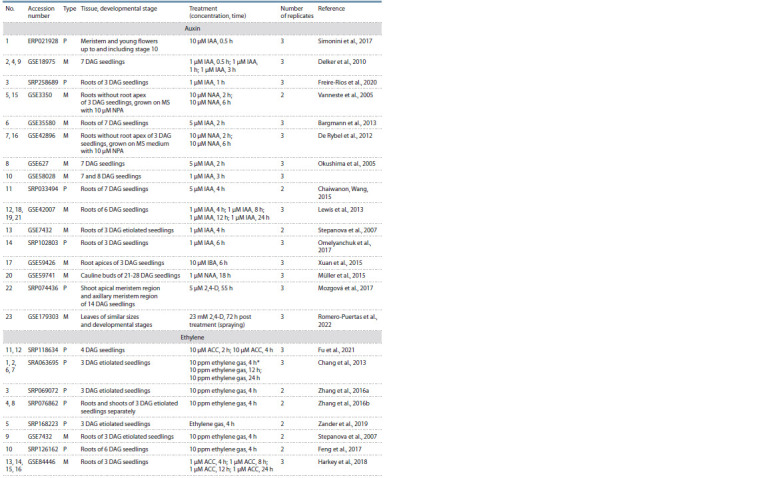
Microarray and RNA-seq data used in this study In experiments No. 1 and 2, ethylene treatment was carried out under the same conditions. The number of biological replicates available for each sample and used for DEG detection is indicated in the sixth column. For DEG calling, untreated control samples collected at the initial time point were used in auxin treatments No. 2, 4, 5, 7, 9, 16, 17, 22, 23, and in ethylene treatments No. 1, 2, 6, 7; otherwise, separate mock treated control samples were employed. R – RNA sequencing; M – microarray experiment; 2,4-D – 2,4-dichlorophenoxyacetic acid; NPA – naphthylphthalamic acid; IAA – indole-3-acetic acid; NAA – 1-naphthaleneacetic acid; IBA – indole 3-butyric acid; DAG – days after germination.

## Results and discussion

In this work, we applied InterTransViewer to comparatively characterize differential gene expression profiles in transcriptional response to phytohormones in A. thaliana. We selected 23 auxin-induced transcriptomes from 16 different studies and 16 transcriptomes induced by ethylene or its precursor ACC from 8 studies (see the Table and Materials and Methods).

**Fig. 2.**
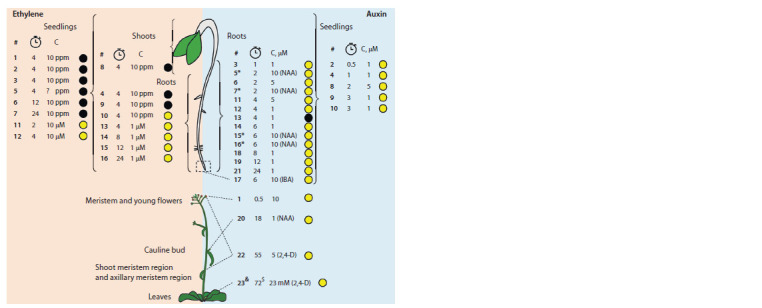
Schematic representation of the experimental conditions, under which transcriptomes selected for comparative analysis were obtained. The asterisk indicates the root segment between the root apical meristem and the root-hypocotyl junction. Ethylene was applied either as a gas (concentration in parts per million) or as its precursor ACC (μM). Auxin was applied as IAA, otherwise indicated. Treatment duration is indicated in hours. Numbers in bold denote the serial numbers of the experiments from Table. C – hormone concentration; black circles – etiolated seedlings; yellow circles – light-grown seedlings; < – spraying; $ – post-treatment; ? – concentration of gaseous ethylene is not given in the primary source.

Figure 2 schematically illustrates the metadata for each transcriptome. It can be seen that despite the similarity of the target factor, the experimental conditions are heterogeneous. In particular, there were differences in the chemical nature of the target factor, its concentration, the method and duration of treatment, the growing conditions of the plants, their age at the time of sample collection, the samples’ nature, and the methods of expression profiling. Only two auxin-induced DEG profiles (No. 9 and 10) from two studies and three ethylene-induced DEG profiles (No. 1, 2, and 3) also from two studies were obtained under similar conditions according to the metadata. Thus, the aim of further comparative analysis was to investigate the homogeneity of phytohormone-induced transcriptomes depending on the conditions under which they were obtained.

Auxin- and ethylene-induced DEG profiles are variable in the number of DEGs

**Fig. 3.**
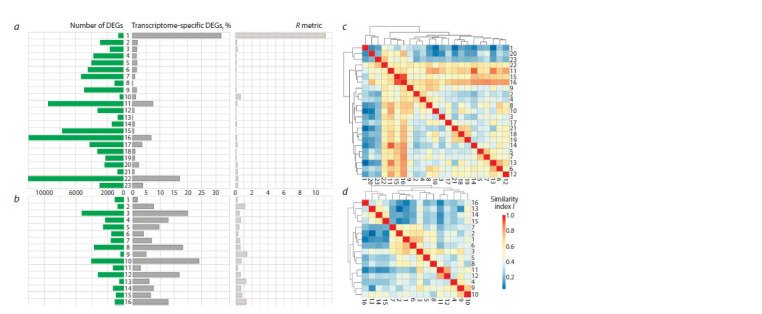
Comparative description of the transcriptional response to auxin and ethylene under different conditions in A. thaliana a, b – number of DEGs and proportion of specific (unique) DEGs in the auxin (a) and ethylene (b) datasets, and R metric for each data set; c, d – pairwise comparison of auxin experiments (c) and ethylene experiments (d ). The similarity index I reflecting the proportion of common DEGs in the smaller list is described in the Materials and Methods section. Experiment serial numbers correspond to those in Table.

First, we characterized each DEG list using the DEGsummary function. Auxin- and ethylene-induced DEG profiles appeared to be heterogeneous in the number of DEGs: ranging from 410 to 11,966 in auxin-induced transcriptomes (median value 3205) and from 379 to 5253 in ethylene-induced ones (median value 1428) (Fig. 3, a, b). The deviation of the DEG numbers from the median value in most cases could be explained by specific experimental conditions. Thus, low numbers of auxinsensitive DEGs were observed in the meristem and young flowers after short-term auxin treatment (No. 1; 586 DEGs) and in the root during long-term treatment (24 h) with low IAA concentration (1 μM) (No. 21, 686 DEGs). The reason for the low number of DEGs in the latter case is because the peak of transcriptional activity changes in response to auxin is observed at 2–8 h of treatment (Lewis et al., 2013). Treatment prolongation up to 12–24 h returns the transcriptional activity of most genes to the level observed in the control (no auxin treatment) samples, and the number of DEGs becomes close to the one detected in short-term (1 h) auxin treatments. A high number of DEGs (No. 22, 11,966 DEGs) was typical for prolonged treatment (55 h) of shoot apices and axillary meristems with 5 μM 2,4-D to induce callus initiation, which is accompanied by significant reprogramming of genome transcriptional activity (Xu et al., 2012). A fairly large number of DEGs was also found in shorter (4–6 h) treatments of seedlings with 5–10 μM IAA, which corresponds to the peak of transcriptional activity changes in response to auxin (Lewis et al., 2013). Notably, a large number of DEGs was observed in transcriptomes of whole roots or roots without root tips, both possessing a wide variety of tissues (No. 11, 15, and 16; 9461, 7692, and 11,905 DEGs, respectively). In the root tip (No. 17), on the contrary, the number of DEGs decreased to 4214, which can be explained by biological homogeneity of the sample (columella, stem cell niche and first progenitors of the initials).

Worth noting is the high value of the R ratio for the DEG profile of meristem and young flowers (No. 1), indicating that this auxin-induced transcriptome has a specific DEG composition compared to all others presented in the study. The reason for a significant deviation of the DEG number from the median in profile No. 10 (410 DEG) could be stress induced by a dramatic change in the seedling cultivation conditions, when the seedlings grown on agarized medium for 6–7 days were placed for a day in liquid medium with constant shaking before auxin treatment. In this case, auxin-sensitive genes associated with the stress response changed their expression both in the experimental and in the control groups. As a result, only genes unrelated to stress manifested as auxin-sensitive DEGs. At the same time, considering the slightly increased value of the R ratio for the DEG list No. 10 compared to the median, we can assume that the quality of these data is not high enough.

A low number of ethylene-sensitive DEGs was observed in roots of light-grown seedlings after a short-term (4 h) treatment with the ethylene precursor, ACC, at a low (1 μM) concentration (No. 13, 522 DEGs), which may be due to the insufficient treatment duration to implement a full response to ethylene. Treatment prolongation up to 8, 12, and 24 hours (No. 14, 15, 16) increased the number of DEGs approximately twofold in all cases (Harkey et al., 2018).

Thus, a complete response to ethylene and the number of DEGs close to the median value were observed for 8-hour and longer treatments. The low number of DEGs in the DEG list No. 9 (379 DEGs) can be linked to technical features of the experiments, given the low number of DEGs in the auxininduced profile No. 13 (657 DEGs) from the same study (Stepanova et al., 2007). Nevertheless, there is no reason to conclude that the quality of these data is low, since the observed deviations are not accompanied by a significant increase in the R ratio value. It is noteworthy that DEG numbers close to the median values were obtained in the experiments implemented with SOLiD RNA sequencing (No. 1, 2, 6, and 7) regardless of the treatment duration (Chang et al., 2013), as well as with Illumina sequencing of shoots (No. 4) and plants of the Ler (Landsberg erecta) ecotype (No. 5), but not Columbia, as in all other cases. In contrast, Illumina sequencing of etiolated shoots and roots yielded the numbers of DEGs greatly exceeding the median value (No. 3, 8, and 10; 5253, 3715, and 4067 DEGs, respectively).

Differential gene expression profiles in response to phytohormones in the samples from different plant parts differ in DEG composition

**Fig. 4.**
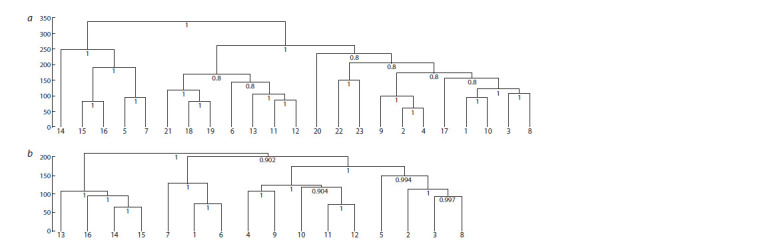
Hierarchical clustering of auxin- (a) and ethylene-induced (b) transcriptomes using the Ward.D2 method.

Next, we investigated the similarity of the DEG lists by DEG composition in more detail. Pairwise comparisons using GetSimMatrix confirmed the specific nature of the transcriptional response to auxin in the shoot meristem and young flowers (profile No. 1) compared to all other organs (see Fig. 3, c). Not surprisingly, a relatively high value of the similarity index for this DEG list (I = 0.47) was observed only with the one of shoot and axillary meristems (No. 22). Next, two groups of similar DEG lists represented the auxin response in whole seedlings and in the roots. The difference between seedling and root DEG profiles was also confirmed with DE_clustering, and it is intuitively clear, since the shoot is represented in the seedling along with the root (Fig. 4). Notably, with the detected intragroup similarity, there was still obvious variability among transcriptomes within each group (see Fig. 2, c). Finally, the DEG lists with more DEGs (No. 11, 15, 16, 22) showed a fairly high similarity index when compared in pairs with all others (see Fig. 3, c).

The qualitative similarity of large DEG lists with each other as well as with smaller DEG lists suggests their validity. Transcriptomic responses cauline leaf buds (No. 20) and leaves (No. 23) showed moderate similarity (I ≥ 0.42) only to the large DEG lists. It can be hypothesized that treatment with high auxin concentrations (No. 11, 15, 16, 22) alters the expression of different groups of genes, each responding to low auxin concentrations only under certain conditions. In addition, the large number of DEGs in the late response may be due to a wide representation of secondary auxin response genes.

Pairwise comparisons of ethylene-induced transcriptomes revealed a discrete group (No. 13–16) from the study by A.F. Harkey et al. (2018) (see Fig. 2, d ). They described gene expression changes in roots after treatment of seedlings grown under continuous light conditions with the ethylene precursor ACC. ACC is also thought to have ethylene-independent biological activity (Vanderstraeten et al., 2019), and light has a significant effect on shaping the transcriptional response to ethylene in A. thaliana (Shi et al., 2016a, b; Luo, Shi, 2019). We hypothesized that the chemical nature of the active compound and the light conditions during seedling growth could act as significant non-target factors in this case. However, DEG lists No. 13–16 showed only moderate similarity to the ones from roots of ethylene-treated seedlings grown under long day (16 h) conditions (No. 10) (I = 0.59, 0.54, 0.52, and 0.49), and were quite different from ACC-induced transcriptomes of whole seedlings grown under 12 h day/12 h night conditions (No. 11 and 12) (0.22 < I < 0.42) (see Fig. 3, d ). Thus, we cannot exclude that the isolation of profiles No. 13–16 may be due to a batch effect. The remaining profiles fell into two groups of similar DEG lists.

The first one included the transcriptional response to ethylene in the roots of seedlings regardless of light conditions, as well as in whole seedlings grown in the presence of light. The second group integrated the response to ethylene in etiolated seedlings or shoots. Thus, we confirmed the known fact that light plays an essential role in shaping the ethylene response (Shi et al., 2016a, b; Luo, Shi, 2019), but additionally we showed that this effect is observed in shoots but not in roots. Notably, hierarchical clustering using log2FC values showed that the time series for ethylene treatment of etiolated seedlings from (Chang et al., 2013) stands out as a separate group (see Fig. 4), which also raises the question of a possible batch effect.

The set of seven ethylene-induced transcriptomes is homogeneous in terms of DEG composition

The number of genes identified as DEGs in a set of transcriptomes (i. e. detected as DEG at least in one of the transcriptomes) essentially depends on the homogeneity of this set. In our case, 20,552 and 10,988 genes were identified as DEGs in at least one auxin- and ethylene/ACC-induced transcriptome, respectively. Given the size of the A. thaliana genome, which contains just over 30,000 genes, this is an unexpectedly large number of DEGs, which is markedly higher than the number of DEGs in individual experiments, and is likely explained by the dependence of transcriptome induction on experimental conditions. Quantification of the homogeneity of the DEG list sets by resampling (using the DE_bootstrap function) expectedly showed their heterogeneity in DEG compositions (Fig. 5, a, b).

At the same time, based on the results of pairwise comparison of DEG lists described in the previous section (see Fig. 3, c, d ), we can suggest the potential homogeneity of auxininduced DEG profiles in the root (No. 5–7, 12–14, 18–21) and ethylene-induced DEG profiles in etiolated seedlings/ shoots (No. 1–3, 5–8). To test this hypothesis, we analyzed the corresponding sets of DEG lists using the DE_bootstrap function. While the set of auxin-induced root transcriptomes still showed heterogeneity (different durations of treatment probably caused differences in DEG composition), no significant differences in the number of ethylene-induced DEGs in etiolated seedlings were found. Thus, the set of ethyleneinduced transcriptomes in etiolated seedlings/shoots (No. 1–3, 5–8), due to their homogeneity, can be reasonably used for meta-analysis (e. g., to better identify weak patterns).

## Conclusion

Meta-analysis of transcriptomic data provides great opportunities for increasing the power of statistical analysis, if the data are homogeneous. However, reasonable selection of experiments for meta-analysis is often hampered by the lack of standards in this field and the absence of convenient software tools for comparative description of DEG lists, in particular, for the construction of user-friendly visualizations. In this work, we proposed a set of quantitative indicators for comparative description of DEG lists (n – number of DEGs; δ – proportion of DEGs specific for a given transcriptome; R – ratio describing the specificity of the transcriptional response; I – similarity index for a pair of transcriptomes based on DEG composition; assessment of the homogeneity of DEG lists) and implemented their calculation and visualization as the InterTransViewer program. We demonstrated that an integrated analysis of the characteristics of individual DEG lists (n, δ, R) in the context of the results of pairwise comparisons of transcriptomes by DEG composition (both using the similarity index I and by clustering based on the fold changes in expression levels) allowed us to range the experiments in the context of each other, to assess the tendency for their integration or segregation, and to generate hypotheses about the influence of significant nontarget factors on the transcriptional response. As a result, this made it possible to identify potentially homogeneous groups of DEG lists.

Subsequent analysis of the homogeneity of these groups using a resampling procedure and the establishment of a significance threshold allowed us to decide whether these data should be used for meta-analysis. Thus, InterTransViewer allows for efficient sampling of the induced transcriptomes depending on the meta-analysis aim and methods.

## Conflict of interest

The authors declare no conflict of interest.
